# pSIG plasmids, MoClo-compatible vectors for efficient production of chimeric double-stranded RNAs in *Escherichia coli* HT115 (DE3) strain

**DOI:** 10.1186/s13007-025-01413-5

**Published:** 2025-07-11

**Authors:** Ching-Feng Wu, Li-Pang Chang, Chan Lee, Ioannis Stergiopoulos, Li-Hung Chen

**Affiliations:** 1 Department of Plant Pathology, National Chung Hsing University, Taichung City, 40227 Taiwan; 2 Advanced Plant and Food Crop Biotechnology Center, National Chung Hsing University, Taichung City, 40227 Taiwan; 3 Department of Plant Pathology, UC Davis, Davis, CA 95616 USA

**Keywords:** RNA interference, Double-stranded RNA, In vivo dsRNA production, *Botrytis cinerea*, *Escherichia coli* HT115 (DE3)

## Abstract

**Background:**

Spray-induced gene silencing (SIGS) is a promising strategy for controlling plant diseases caused by pests, fungi, and viruses. The method involves spraying on plant surfaces double-stranded RNAs (dsRNAs) that target pathogen genes and inhibit pathogen growth via activation of the RNA interference machinery. Despite its potential, significant challenges remain in the application of SIGS, including producing large quantities of dsRNAs for field applications. While industrial-scale dsRNA production is feasible, most research laboratories still rely on costly and labor-intensive in vitro transcription kits that are difficult to scale up for field trials. Therefore, there is a critical need for highly efficient and scalable methods for producing diverse dsRNAs in research laboratories.

**Results:**

This study introduces pSIG plasmids, MoClo-compatible vectors designed for efficient dsRNA production in the *Escherichia coli* RNase III-deficient strain HT115 (DE3). The pSIG vectors enable the assembly of multiple DNA fragments in a single reaction using highly efficient Golden Gate cloning, thereby allowing the production of chimeric dsRNAs to simultaneously silence multiple genes in target pests and pathogens. To demonstrate the efficacy of this system, we generated 12 dsRNAs targeting essential genes in *Botrytis cinerea*. The results revealed that silencing the *Bcerg1*, *Bcerg2*, and *Bcerg27* genes involved in the ergosterol biosynthesis pathway, significantly reduced fungal infection in plant leaves. Furthermore, we synthesized a chimeric dsRNA, Bcergi, that incorporates target fragments from *Bcerg1*, *Bcerg2*, and *Bcerg27*. Nevertheless, the *Bcerg1* dsRNA alone achieved greater disease suppression than the chimeric Bcergi dsRNA.

**Conclusions:**

Here, we developed a highly efficient and scalable method for producing chimeric dsRNAs in *E. coli* HT115 (DE3) in research laboratories using our homemade pSIG plasmid vectors. This approach addresses key challenges in SIGS research, including the need to produce large quantities of dsRNA and identify effective dsRNAs, thus enhancing the feasibility of SIGS as a sustainable strategy for controlling plant diseases and pests in crops.

**Supplementary Information:**

The online version contains supplementary material available at 10.1186/s13007-025-01413-5.

## Background

As global populations rise, the demand for food is projected to increase by 50–60% by 2050 [1]. Pests and pathogens significantly threaten crop production and food security [2], while climate change further exacerbates this issue by potentially facilitating the emergence of new pathogens and expanding their geographical reach [3]. Currently, the primary strategies employed by farmers to combat these threats include the application of chemical pesticides and the development of disease resistant cultivars. However, spraying chemical pesticides poses serious environmental risks, including air, soil and water pollution, and negatively impacts biodiversity and human health [4]. Moreover, the frequent use of pesticides increases the risk of pests and pathogens developing resistance [5–7]. Therefore, a novel approach is needed to ensure sustainable crop protection.

Spray-induced gene silencing (SIGS), which relies on the RNA interference (RNAi) machinery of eukaryotic cells, has proven to be a promising approach for controlling plant diseases caused by insects, fungi, and viruses [8, 9]. This technique involves spraying double-stranded RNAs (dsRNAs) designed to target essential or virulence-associated genes of pathogens onto plant surfaces. Once pathogens absorb these dsRNAs, either directly from the environment or indirectly via plant tissues, the dsRNA induces silencing of their target genes, which ultimately results in suppressing the infection [8, 9]. Given that dsRNAs are sequence-specific and biodegradable, SIGS offers a sustainable and environmentally friendly alternative for disease management. To date, SIGS has been reported to effectively control various phytopathogens, including fungi such as *Botrytis cinerea* and *Fusarium graminearum* [10, 11], viruses such as pepper mild mottle virus and cucumber mosaic virus [12] and insect pests such as the Colorado potato beetle and whitefly [13, 14]. Notably, Calantha™ is the first commercially available SIGS product released by GreenLight Biosciences for controlling the Colorado potato beetle [15].

Despite the potential of SIGS as a sustainable method for controlling plant diseases, several challenges must be addressed before it is effectively utilized in agriculture. Key challenges include the selection of target genes in pathogens or pests that control essential biological or virulence functions, scaling up dsRNA production to quantities that are sufficient for field applications, pathogen uptake of dsRNA and ensuring the environmental stability of dsRNAs [16, 17]. To ensure the effectiveness of SIGS in controlling plant pathogens, recent studies have engineered chimeric dsRNAs against multiple essential or virulence genes, such as those encoding for enzymes involved in ergosterol biosynthesis, cell wall formation, and effector proteins [18–20]. For example, studies on *B. cinerea* have shown that a chimeric dsRNA targeting multiple essential or virulence genes can simultaneously silence these genes, thereby significantly increasing the efficacy of SIGS. Targeted genes in *B. cinerea* included those in the RNAi pathway, such as *dicer1* and *dicer2* [11], as well as *erg1*, *erg11*, and *erg13*, which are vital for ergosterol biosynthesis [18]. Likewise, a chimeric dsRNA targeting the *cyp51A*, *cyp51B*, and *cyp51C* genes has been shown to effectively control *F. graminearum* infections in wheat [10]. These examples highlight the effectiveness of using chimeric dsRNAs to simultaneously silence multiple genes in pathogens. However, producing chimeric dsRNAs by assembling multiple DNA fragments into a plasmid via traditional cloning methods can be a complex and challenging task. Thus, a more streamlined and efficient method for producing chimeric dsRNAs is needed to facilitate the practical application of SIGS in disease control.

Currently, most studies utilize in vitro transcription kits to produce dsRNAs, but these kits are expensive, labor intensive, and unsuitable for scaling up. To meet the demands for large-scale dsRNA production that is required for SIGS, an in vivo method based on using the RNase III-deficient *E. coli* HT115 (DE3) strain offers a cost-effective, scalable, and adaptable alternative. RNase III is a nuclease that specifically degrades dsRNAs, and its inactivation in the HT115 (DE3) strain allows for the production of dsRNAs. Additionally, the introduction of a plasmid with two inverted IPTG-inducible T7 RNA polymerase promoters [21] in the *E. coli* HT115 (DE3) strain enables the transcription of two complementary single-stranded RNAs (ssRNAs) upon IPTG induction. The ssRNAs then hybridize in the cytosol to form the desired dsRNA, while unhybridized ssRNAs are degraded by cytosolic RNases [22].

*E. coli* HT115 (DE3) and the dsRNA-producing plasmid L4440 were pivotal in early RNAi research in nematodes, facilitating gene function studies [23, 24]. Furthermore, the T444T plasmid, featuring two T7 polymerase termination sequences adjacent to the T7 promoter, has enhanced RNAi efficiency in *Caenorhabditis elegans* [25]. Additional systems, such as pET28-BL21(DE3) RNase III and the bacteriophage phi6-*Pseudomonas syringae* system, have also improved in vivo dsRNA production [26]. Despite these advancements, there remains a significant gap in the availability of high-efficiency dsRNA production systems capable of effectively screening target genes in pathogens.

A variety of cloning techniques, including Golden Gate (GG) [27], Gibson Assembly (GA) [28], and sequence and ligation independent cloning (SLIC) [29], have been developed to assemble multiple DNA fragments efficiently and seamlessly in a highly efficient manner. Notably, GG-based modular cloning (MoClo) toolkits have become prominent tools in plant genome editing and synthetic biology [30, 31]. Additionally, the pRNAi-GG vector facilitates the construction of intron-containing hairpin RNAs capable of silencing multiple genes simultaneously within plants [32, 33]. GG assembly utilizes Type IIS restriction enzymes, which cleave DNA outside their recognition sequences, creating overhangs. These overhangs can be specifically designed to allow for the assembly of multiple DNA fragments into a final vector. The absence of restriction enzyme recognition sequences in the final construct enables the digestion and ligation reactions to occur concurrently within the same tube, streamlining the cloning process and enhancing throughput [27].

In this study, we present a MoClo-compatible pSIG plasmid vector system designed for the efficient production in *E. coli* HT115 (DE3) of chimeric dsRNAs targeting multiple genes in pests and pathogens of interest. The pSIG vectors feature two inverted T7 promoters and terminators to facilitate dsRNA synthesis upon IPTG induction. Additionally, a *SacB* gene placed between the T7 promoters serves as a negative selection marker to ease the selection of positive transformants. Our results demonstrate that the cloning efficiency reached nearly 100% for single-fragment constructs and 60% for three-fragment chimeric constructs when using the pSIG1 vector. The system allows the entire workflow, from plasmid construction to dsRNA production in *E. coli* HT115 (DE3), RNA extraction, and dsRNA purification, to be completed within four days. We employed this system to synthesize dsRNAs targeting genes involved in the biosynthesis of ergosterol, chitin, and melanin in *B. cinerea*. The application of dsRNAs against *Bcerg1, Bcerg2,* and *Bcerg27* significantly reduced lesion sizes of *B. cinerea* on *Nicotiana benthamiana* leaves. We next generated a chimeric dsRNA, Bcergi, engineered to simultaneously target *Bcerg1*, *Bcerg2*, and *Bcerg27*. Unexpectedly, the *Bcerg1* dsRNA alone achieved more effective suppression of *B. cinerea* than the chimeric RNA. Overall, the pSIG system offers a highly efficient and scalable approach for producing chimeric or single-fragment dsRNAs in *E. coli* HT115 (DE3), thereby enhancing the practical application of SIGS in disease control.

## Results

### Construction of pSIG plasmids for chimeric dsRNA production in *E. coli* HT115 (DE3)

**Fig. 1:**
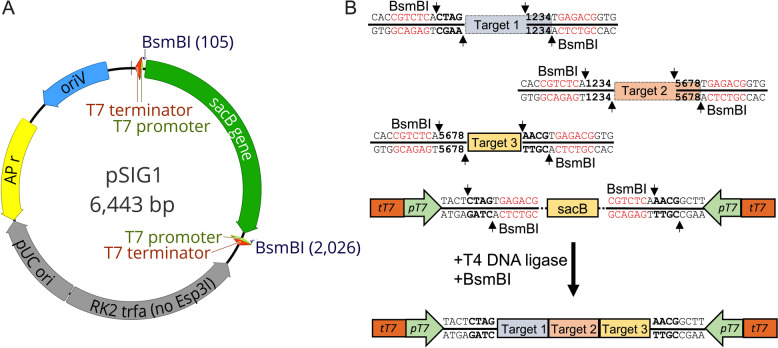
Schematic of the pSIG1 plasmid vector and the Golden Gate (GG) strategy used to assemble multiple DNA fragments into a single chimeric construct. **A** Diagram of the pSIG1 plasmid vector. The plasmid contains two T7 promoters, two T7 terminators, and BsmBI recognition sites, along with a *SacB* gene expression cassette cloned into the backbone of pICH47732. Construction was achieved via GG cloning. **B** GG strategy for assembling three DNA fragments into a single chimeric construct in the pSIG1 plasmid. Each DNA fragment corresponds to a different gene targeted by dsRNAs. The fragments are PCR-amplified using primers that include gene-specific sequences, BsmBI recognition sites, and adapter sequences. These adapters facilitate seamless ligation by ensuring compatibility with either the pSIG1 vector or the other DNA fragments. The numbers on the fragments indicate the custom sequence. The assembly is performed in a single GG reaction with BsmBI and T4 DNA ligase

To establish a highly efficient method for producing chimeric dsRNAs in *E. coli* HT115 (DE3), we employed the Golden Gate (GG) cloning strategy to construct the pSIG plasmids. Specifically, pSIG1 and pSIG2 plasmids were designed using the pICH47732 vector within the MoClo system [30]. The constructs include a *SacB* gene cassette flanked by two BsmBI type IIS restriction enzyme recognition sites in inverted orientation, along with two inverted T7 promoters and terminators (Fig. 1A and Supplementary Fig.  1 A). The *SacB* gene cassette, derived from pK18mobSacB, serves as a negative selectable marker to increase the selection of pSIG plasmids with cloned fragments [34]. The presence of two BsmBI sites enables the assembly of multiple DNA fragments in a single reaction through GG cloning. Following fragment assembly, the dsRNA-producing pSIG plasmid is transformed into the *E. coli HT115* (DE3) strain, which harbors an IPTG-inducible T7 RNA polymerase and an inactivated RNase III nuclease. The T7 RNA polymerase drives transcription of the DNA fragments by binding to the two inverted T7 promoters positioned on their opposite sides, thereby leading to the synthesis of sense and antisense RNA strands that hybridize to form the desired dsRNAs. The adjacent T7 terminators serve to block the transcription of nonspecific RNA fragments from the vector backbone, thereby improving the selectivity of dsRNA production in the *E. coli* [25].

To assemble multiple DNA fragments into pSIG plasmids, the targeted gene regions in fungi are PCR amplified using primers that include BsmBI recognition and cleavage sites at their N-terminus. The resulting PCR products and pSIG plasmid are next digested with BsmBI and ligated using T4 DNA ligase in a single GG reaction. Following the integration of the target fragments into the pSIG plasmid, the BsmBI recognition sites are eliminated, enabling the selection of successfully assembled plasmids through *SacB*-driven negative selection after transformation into the *E. coli* HT115 (DE3) strain (Fig. 1B and Sup. 1B). The primary difference between pSIG1 and pSIG2 is in the number of extra base pairs in the resulting dsRNAs. dsRNAs derived from pSIG1 include an additional 20 base pairs from the plasmid backbone and T7 promoter, whereas dsRNAs from pSIG2 contain only an additional two base pairs from the T7 promoter (Fig. 1B and Supplementary Fig. 1B).

### Validation of the negative selection and cloning efficiency of the pSIG1 plasmid

**Fig. 2:**
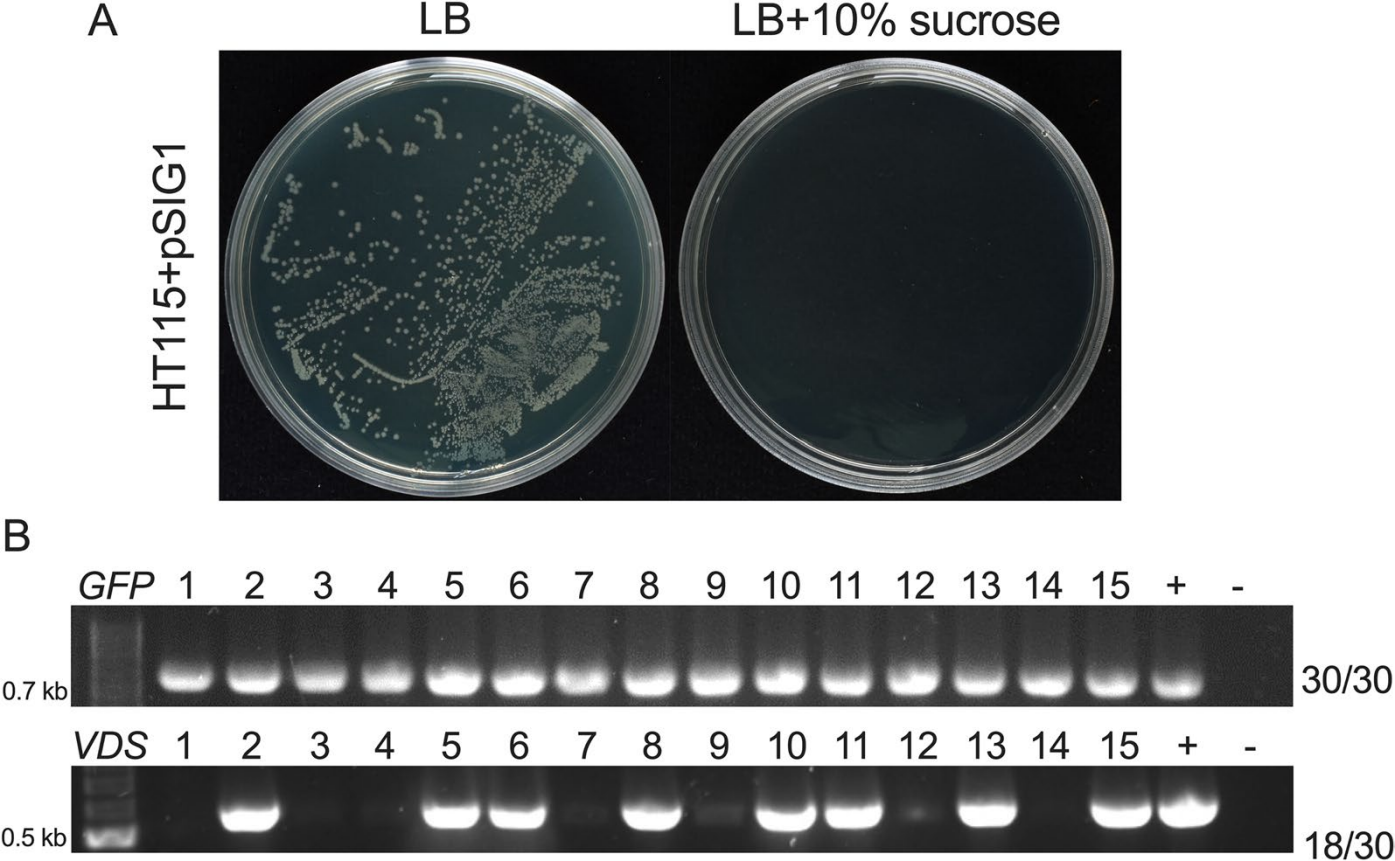
Negative selection and cloning efficiency in the pSIG1 plasmid vector. **A** Lethality induced by the *SacB* gene in the pSIG1 plasmid. *E. coli* HT115 (DE3) cells harboring the pSIG1 plasmid were spread on LB agar plates with or without 10% sucrose. Growth inhibition on sucrose-containing plates confirmed effective negative selection mediated by the *SacB* gene. **B** Cloning efficiency of the pSIG1 plasmid for single and multiple DNA fragments. A single 717 bp DNA fragment from the GFP gene, and a 516 bp VDS DNA fragment composed of 169 bp, 179 bp, and 168 bp DNA fragments from the *VPS51, DCTN1,* and *SAC1* genes, respectively, of *Botrytis cinerea*, were inserted into the pSIG1 plasmid via Golden Gate (GG) cloning. The GG reaction products were transformed into the *E. coli* HT115 (DE3) strain and selected on LB plates supplemented with 10% sucrose. Cloning efficiency was evaluated using colony PCR by analyzing 30 colonies from three independent experiments for each condition

To assess the effectiveness of the *SacB* gene as a negative selection marker, the empty pSIG1 plasmid vector was transformed into *E. coli* HT115 (DE3) and cultured on LB agar with or without 10% sucrose (Fig. 2A). Growth of *E. coli* HT115 (DE3) containing the empty pSIG1 plasmid vector was completely inhibited on LB agar supplemented with 10% sucrose, while growth was observed on LB agar without sucrose. This confirmed that the *SacB* gene efficiently selects against *E. coli* HT115 (DE3) carrying the empty pSIG1 plasmid in the presence of 10% sucrose.

We next evaluated the cloning efficiency of single DNA fragments into the pSIG1 vector. A 717 bp DNA fragment of the *GFP* gene was PCR-amplified and cloned into pSIG1 via GG cloning. The reaction mixture was transformed into *E. coli* HT115 (DE3) and positive transformants were selected on LB agar supplemented with 10% sucrose. Colony PCR analysis of 30 randomly chosen colonies revealed that all contained the correct pSIG1-GFP construct (Fig. 2B), demonstrating 100% cloning efficiency for a single fragment. Next, we assessed the cloning efficiency of three DNA fragments into the pSIG1 plasmid vector by generating VDS dsRNA, a dsRNA previously shown to suppress *B. cinerea* disease symptoms in a range of host tissues [35]. The 516 bp VDS fragment included a 169 bp fragment of the *VPS51* gene (*V*), a 179 bp fragment of the *DCTN1* gene (*D*), and a 168 bp fragment of the *SAC1* gene (*S*). All fragments were PCR amplified using *B. cinerea* genomic DNA as a template. After GG cloning into pSIG1, colony PCR analysis indicated a 60% cloning efficiency, with 18 of the 30 colonies containing all three fragments correctly assembled into a single *VDS-*chimeric construct positioned between the inverted T7 promoters in the pSIG1 vector (Fig. 2B). Collectively, these results demonstrate that pSIG1, in combination with GG cloning, provides a high-efficiency platform for constructing dsRNA-producing plasmids in the *E. coli* HT115 (DE3) strain.

### Evaluation of dsRNA production and purification efficiency

**Fig. 3:**
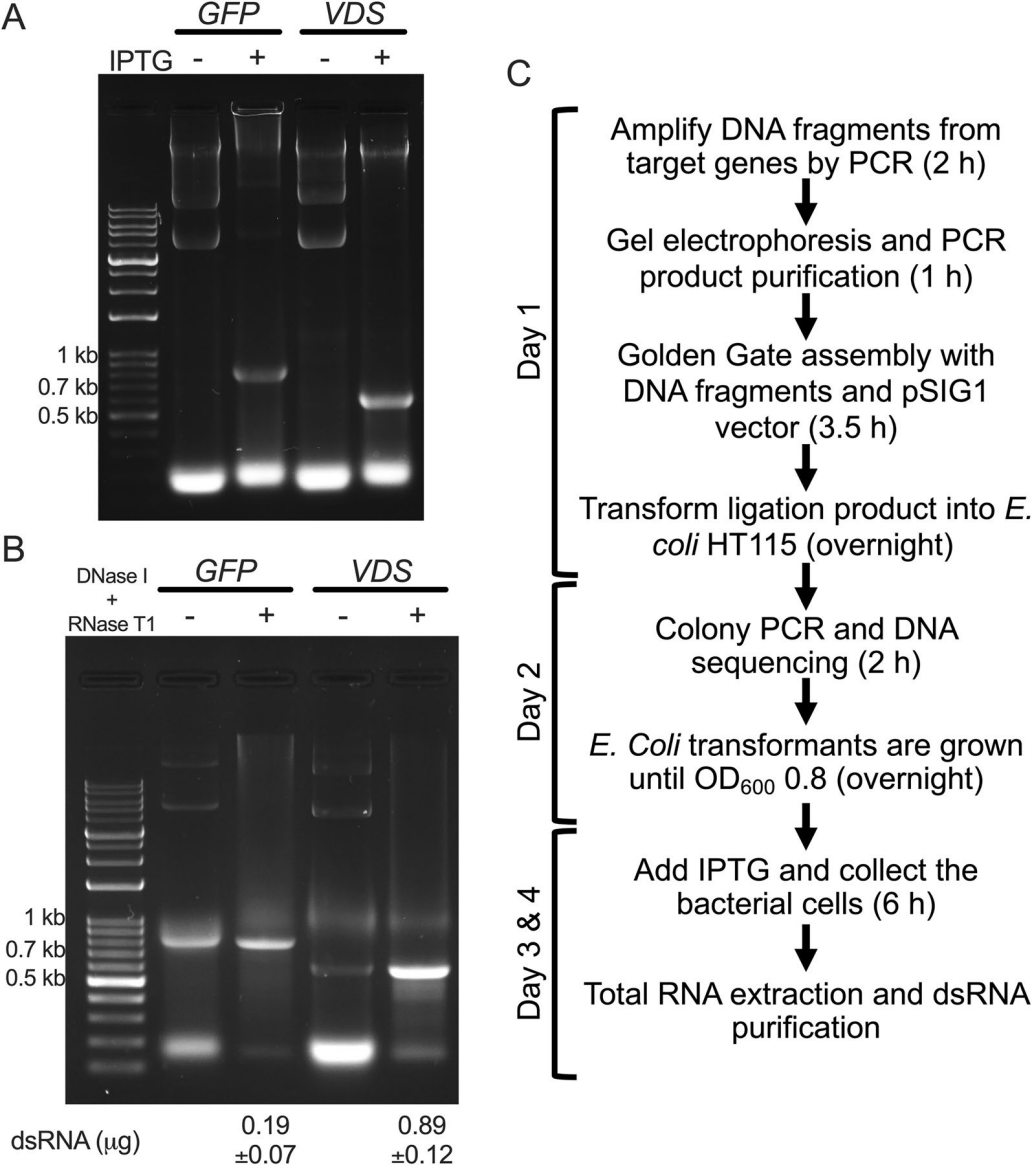
Production and purification of dsRNA via the pSIG1 plasmid in *E. coli* HT115 (DE3). **A** Total RNA extraction with or without IPTG induction. Total RNA was extracted from *E. coli* HT115 (DE3) cultures containing the pSIG1 plasmid using Trizol (TRI) Reagent. Gel electrophoresis showed the presence of dsRNAs corresponding to the GFP (737 bp) and VDS (536 bp) gene fragments following IPTG induction, whereas no dsRNA was detected in the uninduced samples. **B** Purification of dsRNA via DNase I and RNase T1 treatment. Total RNA was enzymatically treated to digest DNA and single-stranded RNA (ssRNA). Posttreatment samples presented a reduced intensity of DNA bands (upper gel region) and ssRNA bands (lower gel region), with the dsRNA bands remaining intact. **C** Timeline of dsRNA production. Schematic representation of the production and purification process for dsRNA via the pSIG1 system in the *E. coli* HT115 (DE3) strain

Following the construction of the pSIG1-GFP and pSIG1-VDS plasmids, they were transformed into the *E. coli* HT115 (DE3) strain, and dsRNA production was induced by IPTG. Total RNA was extracted via the Trizol™ method. Upon IPTG induction, GFP dsRNA (737 bp) and VDS dsRNA (536 bp) were clearly detectable on an agarose gel, whereas no dsRNA bands were observed in the absence of IPTG treatment (Fig. 3A). To ensure the purity of the dsRNAs, the extracted total RNA was treated with DNase I to remove DNA and RNase T1 to degrade ssRNA. The dsRNA was subsequently purified and concentrated using a filter-based RNA clean-up kit, and the resulting pure dsRNA was visualized on an agarose gel (Fig. 3B). The concentrations of GFP dsRNA and VDS dsRNA were 0.19 ± 0.07 µg mL⁻^1^ and 0.89 ± 0.12 µg mL⁻^1^ of bacterial culture, respectively. Overall, this high-efficiency system enables the rapid and streamlined process of plasmid construction, dsRNA production, and dsRNA purification within four days, yielding pure dsRNA that is ready for use in downstream applications (Fig. 3C).

### The pSIG1 plasmid is compatible with the MoClo toolkit

**Fig. 4:**
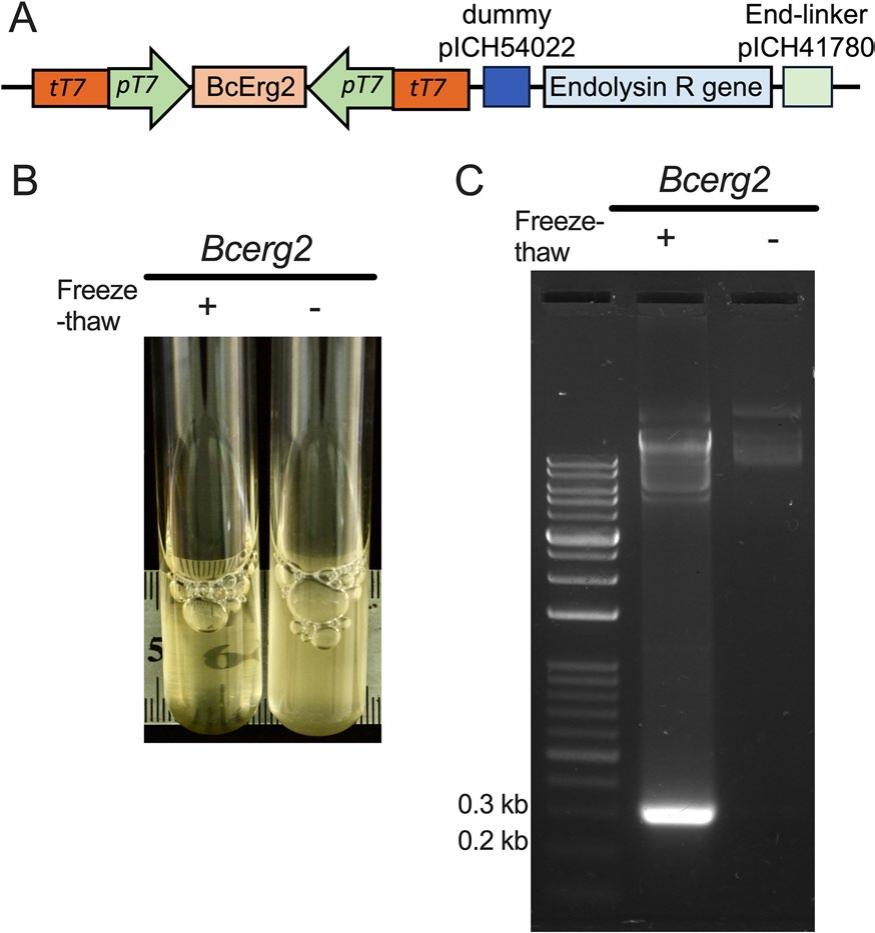
Compatibility of the pSIG1 plasmid with the MoClo system for generating autolytic *E. coli* following a freeze‒thaw cycle. **A** Schematic of the plv2-dsErg2-R plasmid. The *Bcerg2*-targeting dsRNA expression cassette, the endolysin *R* gene, a dummy sequence, and an end-linker were assembled into the MoClo Level 2 plasmid pAGM4723 via Golden Gate cloning. **B** Bacterial autolysis induced by a freeze‒thaw cycle. Cultures of *E. coli* HT115 (DE3) carrying the plv2-dsErg2-R plasmid became transparent after a freeze‒thaw cycle, indicating cell lysis. **C** Release of *Bcerg2*-targeting dsRNA from autolyzed *E. coli*. Nucleotides from the supernatant of freeze-thawed bacterial cultures were concentrated and analyzed using gel electrophoresis. A distinct 270 bp *Bcerg2* dsRNA band was detected

The pSIG plasmids were derived from the MoClo level 1 vector pICH47732. Consequently, the dsRNA expression cassette in the pSIG plasmids can be seamlessly integrated into MoClo level 2 plasmids via GG cloning. To assess the assembly of multiple fragments and subsequent dsRNA production in the level 2 vector, we constructed the plasmid plv2-dsErg2-R, which incorporates a dsRNA expression cassette targeting the *B. cinerea erg2* gene and a phage endolysin *R* gene that encodes a phage enzyme capable of degrading bacterial cell walls. First, a 250 bp fragment of the *Bcerg2* gene was cloned into pSIG1, while the endolysin *R* gene was inserted into the pICH47742 vector. Using GG cloning with the restriction enzyme BpiI, we successfully assembled the dsRNA expression cassette, the endolysin *R* gene, a dummy fragment (pICH54022), and an end-linker (pICH41780) into the MoClo level 2 vector pAGM4723. The fragment orientation within the resulting plv2-dsErg2-R plasmid is shown in Fig. 4A.

To assess the functionality of the endolysin *R* gene, the *E. coli* HT115 (DE3) strain was transformed with the plv2-dsErg2-R plasmid. During bacterial growth, endolysin R accumulates in the cytosol but upon freeze-thawing, the weakened cell membrane allows endolysin R to enter the periplasmic space, leading to cell lysis [36, 37]. To examine autolysis through freeze-thawing, *E. coli* HT115 (DE3) carrying plv2-dsErg2-R was cultured in LB media supplemented with 1 mM IPTG, then frozen at − 80 °C for 30 min, and finally incubated at 37 °C for 90 min with shaking at 100 rpm. After this freeze–thaw cycle, the culture became transparent, indicating successful cell lysis (Fig. 4B). Next, we evaluated the release of dsRNA after bacterial autolysis. To do this, the bacterial cultures with and without freeze-thawing were centrifuged, and the supernatant was purified using an RNA clean-up kit. Agarose gel analysis confirmed the presence of *Bcerg2-*targeting dsRNA in the supernatant only following cell lysis after a freeze–thaw cycle (Fig. 4C).

In summary, these results highlight the compatibility of the pSIG1 plasmid with the MoClo toolkit. Both level 1 and level 2 plasmids containing dsRNA expression cassettes enable the efficient production of diverse dsRNAs in the *E. coli* HT115 (DE3) strain.

### Silencing of genes in the ergosterol biosynthesis pathway of *Botrytis cinerea* reduces disease incidence

**Fig. 5:**
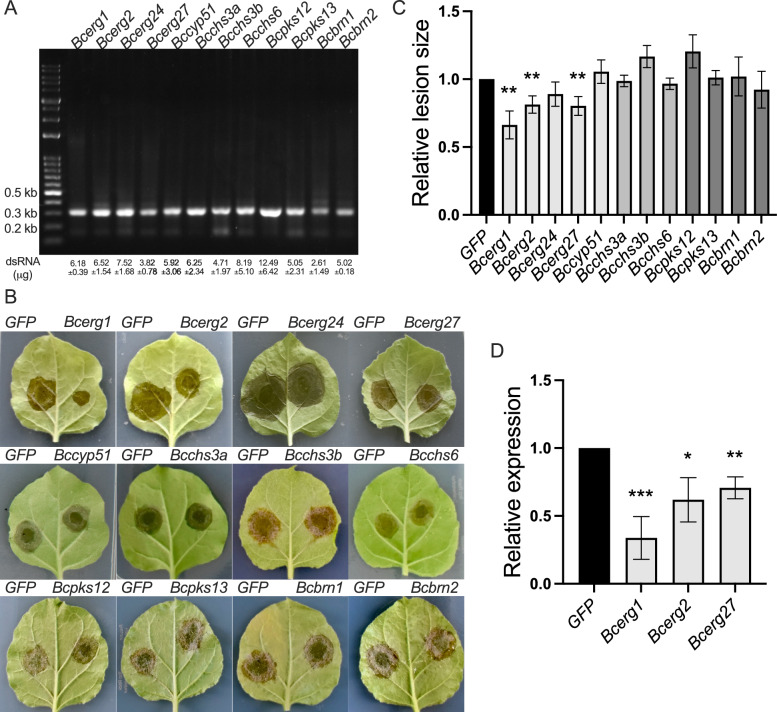
dsRNAs targeting certain genes in the ergosterol biosynthesis pathway, reduce *Botrytis cinerea* infections. **A** Production and extraction of dsRNAs from *E. coli* HT115 (DE3). Twelve dsRNAs targeting essential fungal genes in the ergosterol, chitin, and melanin biosynthesis pathways were produced in *E. coli* HT115 (DE3). The dsRNAs were extracted using the Trizol (TRI) Reagent and purified with DNase I and RNase T1. All the dsRNAs displayed the expected size (~ 270 bp) on an agarose gel. **B** Co-inoculation of dsRNAs and *B. cinerea* spores on *Nicotiana benthamiana* leaves. *Botrytis cinerea* spores (100 spores) were mixed with dsRNAs with a final volume of 20 μL and applied to the right side of detached leaves. GFP dsRNA was applied to the left side as a control. Each treatment was performed at least three times. **C** Relative lesion size following *B. cinerea* infection. Lesion sizes were measured at four days post-inoculation (dpi). The lesion size from the GFP-targeting dsRNA control was set to 1, and the relative lesion sizes were calculated. The error bars represent the SEMs of three biological replicates with more than three technical repeats. Statistical significance was determined by Student’s t test (**P < 0.01). **D** Relative expression levels of *Bcerg1*, *Bcerg2*, and *Bcerg27* measured via qRT‒PCR. Gene expression levels were normalized to *Bcactin* expression. The error bars represent the SEMs of three biological replicates. Statistical significance was calculated via Student’s t test (*P < 0.05, **P < 0.01, ***P < 0.001)

Once the pSIG dsRNA production system was established, we generated 12 dsRNAs targeting essential genes in *B. cinerea*. Target genes included genes involved in ergosterol biosynthesis (*Bcerg1*, *Bcerg2*, *Bcerg24*, *Bcerg27*, and *Bccyp51*), chitin biosynthesis (*Bcchs3a*, *Bcchs3b*, and *Bcchs6*), and melanin biosynthesis (*Bcpks12*, *Bcpks13*, *Bcbrn1*, and *Bcbrn2*). These genes were selected on the basis of their relevance to fungicide target sites, as categorized by the FRAC mode of action classification [38] (Table 1). To assess RNAi efficiency and potential off-target effects in humans, we analyzed the 12 dsRNAs using the si-Fi software [39]. The analysis indicated that only the *Bcerg1* dsRNA had potential off-target interactions with the human genome (Table 1). Each 250 bp DNA fragment corresponding to these genes was amplified from *B. cinerea* cDNA and cloned into the pSIG1 plasmid. Subsequently, all 12 dsRNAs were successfully produced in *E. coli* HT115 (DE3) and purified via DNase I and RNase T1 treatments. From 1 mL bacterial culture, the amount of dsRNA is in the range of 2.61 to 12.49 µg (Fig. 5A). The dsRNA concentrations were further measured and normalized to ensure uniform quantities for use in subsequent experiments.

**Table 1:** List of *Botrytis cinerea* gene targets

Gene	Protein function	FRAC mode of action	NCBI accession numbers	Off-target (gene)
*erg1*	squalene epoxidase	G: Sterol biosynthesis in membranes	XM_001547426.2	1
*erg2*	△8 → △7-isomerase	G: Sterol biosynthesis in membranes	XM_001558404.2	0
*erg24*	△14-reductase	G: Sterol biosynthesis in membranes	XM_024695680.1	0
*erg27*	3-keto reductase	G: Sterol biosynthesis in membranes	XM_024691999.1	0
*cyp51*	C14-demethylase	G: Sterol biosynthesis in membranes	XM_001549911.2	0
*chs3a*	chitin synthase III	H: Cell wall biosynthesis	AF494188.1	0
*chs3b*	chitin synthase III	H: Cell wall biosynthesis	AF529208.1	0
*chs6*	chitin synthase VI	H: Cell wall biosynthesis	AY515144.1	0
*pks12*	polyketide synthase	I: Melanin synthesis in cell wall	XM_024691604.1	0
*pks13*	polyketide synthase	I: Melanin synthesis in cell wall	XM_001547045.2	0
*brn1*	trihydroxynaphthalene reductase	I: Melanin synthesis in cell wall	KT726848.1	0
*brn2*	trihydroxynaphthalene reductase	I: Melanin synthesis in cell wall	XM_024692191.1	0

FRAC: Fungicide Resistance Action Committee

To assess the impact of the 12 dsRNAs on *B. cinerea* virulence, we inoculated *N. benthamiana* leaves with *B. cinerea* spores in the presence of the dsRNAs. Each dsRNA was tested individually, and as a negative control, GFP-targeting dsRNA was co-inoculated with spores on the opposite side of the same leaf. At four days post fungal inoculations, lesion sizes were measured (Fig. 5B, C). Compared with the GFP-targeting dsRNA control, treatment with dsRNAs targeting the *Bcerg1*, *Bcerg2*, and *Bcerg27* genes resulted in significant reductions in lesion size by 34%, 19%, and 20%, respectively (Fig. 5C). Gene silencing of *Bcerg1*, *Bcerg2*, and *Bcerg27* was confirmed via RT-qPCR at three days post inoculation (Fig. 5D). Interestingly, dsRNAs targeting the ergosterol biosynthesis pathway significantly reduced *B. cinerea* infection, whereas dsRNAs targeting the chitin and melanin biosynthesis pathways had no noticeable effect on lesion size in *N. benthamiana* leaves. In summary, our results demonstrate that dsRNAs produced in *E. coli* HT115 (DE3) strain using pSIG plasmids can effectively facilitate targeted gene silencing, offering a promising strategy for controlling *B. cinerea* through SIGS.

### Chimeric dsRNA targeting three ergosterol-biosynthesis genes does not surpass *Bcerg1* dsRNA in suppressing *Botrytis cinerea*

**Fig. 6:**
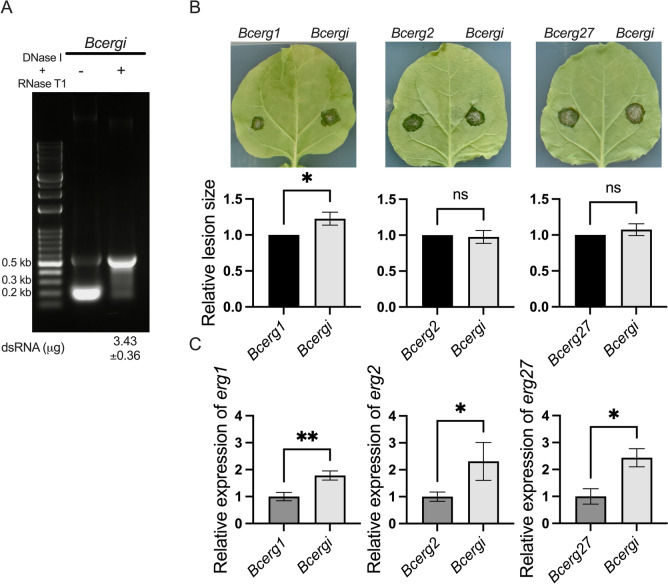
A *Bcerg1* dsRNA suppresses *Botrytis cinerea* infection more effectively than a 470 bp chimeric dsRNA targeting three ergosterol-biosynthesis genes. **A** Production and extraction of the chimeric dsRNA from *E. coli* HT115 (DE3). A 470 bp chimeric dsRNA composed of 150 bp fragments from *Bcerg1*, *Bcerg2*, and *Bcerg27* was expressed in *E. coli* HT115 (DE3), extracted with TRIzol, and treated sequentially with DNase I and RNase T1. Agarose gel electrophoresis revealed a single band at the expected size (470 bp). **B** Co-inoculation of dsRNAs and *B. cinerea* spores on *Nicotiana benthamiana* leaves. *Botrytis cinerea* spores (100 spores) were mixed with either *Bcerg1*, *Bcerg2*, *Bcerg27* or the chimeric Bcergi dsRNA and applied to opposite halves of the same leaf. Lesion areas were measured 4 days post-inoculation (dpi). Lesions from the single-gene dsRNA treatments were normalized to 1, and the relative lesion sizes for the chimeric dsRNA are shown. The error bars represent the SEMs of three biological replicates with more than three technical repeats. Statistical significance was conducted with Student’s t test (**P < 0.01). **C** Relative expression levels of *Bcerg1*, *Bcerg2*, and *Bcerg27* measured via qRT-PCR. Gene expression levels were normalized to *Bcactin* expression. The error bars represent the SEMs of three biological replicates. Statistical significance was calculated via Student’s t test (*P < 0.05, **P < 0.01, ***P < 0.001)

To explore whether targeting multiple ergosterol biosynthesis genes could enhance the inhibitory effect, we constructed the pSIG1-Bcergi plasmid to produce a chimeric dsRNA (470 bp) composed of 150 bp fragments from *Bcerg1*, *Bcerg2*, and *Bcerg27* in *E. coli* HT115 (DE3) strain. After DNase I and RNase T1 treatment, purified Bcergi dsRNA was obtained (Fig. 6A), yielding approximately 3.43 ± 0.36 µg per 1 mL of bacterial culture. The inhibitory efficacy of Bcergi dsRNA was evaluated through pairwise co-inoculation experiments. Fungal conidia were co-inoculated with Bcergi dsRNA on one side of the leaf, while the opposite side received dsRNA targeting either *Bcerg1*, *Bcerg2*, or *Bcerg27*. Four days after inoculation, Bcergi dsRNA exhibited inhibition comparable to that of *Bcerg2* and *Bcerg27* dsRNAs. In contrast, *Bcerg1* dsRNA showed a superior inhibitory effect compared to Bcergi (Fig. 6B). The RT-qPCR showed that each 270 bp dsRNA reduced its corresponding gene expression more effectively than the 470 bp chimeric dsRNA (Fig. 6C). These findings indicate the chimeric dsRNA may not inherently enhance silencing efficiency or disease control. Instead, targeting a single essential gene, particularly *Bcerg1*, can achieve superior inhibition.

## Discussion

In this study, we developed a high-efficiency and scalable dsRNA production system using pSIG plasmid vectors and the *E. coli* HT115 (DE3) strain. The system demonstrated high efficiency in assembling multiple DNA fragments via a one-step GG reaction, enabling the production of chimeric dsRNAs that simultaneously target multiple genes in pathogens. Using this approach, we successfully produced 12 dsRNAs targeting essential genes in *B. cinerea*. Our findings highlighted that genes involved in the ergosterol biosynthesis pathway such as *Bcerg1*, *Bcerg2*, and *Bcerg27*, are promising targets for controlling this pathogen. Therefore, the system represents a viable alternative to traditional in vitro dsRNA production methods and has significant potential for advancing RNA-based disease management methods.

The use of the GG method for pSIG plasmid assembly is notable for its simplicity and efficiency. GG cloning is widely utilized in synthetic biology and genome editing, particularly in plant sciences [30, 31]. However, several challenges remain, including the need for effective selection markers to distinguish recombinant plasmids from empty ones. While positive selection markers such as lacZ and red fluorescence proteins are commonly used, the application of lacZ requires additional reagents such as X-gal and IPTG [30]. Negative selection markers, such as the *ccdB* gene, are also employed but necessitate the use of resistant strains for vector propagation [40]. In this study, we employed the *SacB* gene as a negative selection marker, which enabled the efficient counter-selection of *E. coli* HT115 (DE3) carrying empty pSIG plasmids when grown on sucrose-containing media [34, 41]. The pSIG system demonstrated high efficiency for single DNA fragments, as evidenced by the successful cloning of 12 *B. cinerea* gene fragments into the pSIG1 plasmid, thus streamlining the systematic screening of essential fungal genes for further applications.

The pSIG system enables the production and purification of dsRNAs in *E. coli* HT115 (DE3) within a four-day timeframe. Following purification using RNase T1 and DNase I, the concentration of *Bcerg1* dsRNA reached approximately 6.18 ± 0.39 µg per 1 mL bacterial culture. Compared to in vitro transcription kits such as the MEGAscript™ RNAi Kit, which yields 50 ~ 100 µg per reaction, this in vivo system offers significant advantages in terms of scalability. For example, a culture volume of less than 20 mL can yield more than 100 µg of *Bcerg1* dsRNA. Given that approximately 9.9 g of dsRNAs per hectare are required to control the Colorado potato beetle during field tests [13], our system can produce sufficient dsRNAs for small field tests (100 m^2^) via a standard 20 L benchtop bioreactor. In contrast, achieving the same output through in vitro synthesis would require nearly 1000 reactions, making it impractical for large-scale applications. Although commercial cell-free systems offer lower costs for large dsRNA orders (less than $1/g) [42], they often impose minimum order quantities. Thus, the pSIG system provides an alternative and scalable method for producing diverse dsRNAs in a laboratory setting, enabling initial screening of candidates before committing to large-scale production.

One of the primary costs associated with in vivo dsRNA production is total RNA extraction via TRIzol™ reagent, as well as dsRNA purification via DNase I and RNase T1 treatment. Alternative approaches, such as the RNAswift method, have been introduced to reduce costs by using readily available reagents such as ethanol, isopropanol, and SDS for nucleotide extraction [43]. Furthermore, Niño-Sánchez et al. demonstrated that living bacteria or bacterial lysates containing dsRNAs against genes in *B. cinerea* result in their efficient silencing, suggesting that dsRNA purification may not always be necessary for further applications [37]. Collectively, these findings underscore the potential of the pSIG system as a scalable and cost-efficient solution for producing dsRNAs for small-scale SIGS field trials.

The pSIG vectors are Level 1 plasmids derived from MoClo toolkits, thereby enabling the dsRNA-producing cassette to be assembled with other genes or DNA fragments into a Level 2 vector via GG cloning. For example, we successfully combined a *Bcerg2*-targeting dsRNA cassette with the phage endolysin *R* gene, a phage enzyme for degrading bacterial cell walls, into a Level 2 plasmid. Upon freeze-thawing, *E. coli* HT115 (DE3) harboring this plasmid autolyzed, releasing the *Bcerg2*-targeting dsRNA into the culture medium. This modularity enables the coproduction of other functional proteins, including viral proteins such as tombusvirus p19, which has previously been used to produce siRNAs in *E. coli* [44]. This versatility could enhance the efficacy of SIG-based disease control strategies.

Our results demonstrate that dsRNAs targeting ergosterol biosynthesis genes, such as *Bcerg1*, *Bcerg2*, and *Bcerg27*, effectively reduce *B. cinerea* infection in *N. benthamiana* leaves, which is consistent with previous studies showing the efficacy of ergosterol biosynthesis-targeting dsRNAs against *B. cinerea* [18], *F. graminearum* [10], and *Golovinomyces orontii* [19]. In contrast, dsRNAs targeting the chitin and melanin biosynthesis pathways did not significantly inhibit *B. cinerea* infection. These findings align with reports that naked dsRNAs targeting chitin synthesis genes fail to reduce disease symptoms in *B. cinerea* [45] and *Sclerotinia sclerotiorum* [46], although several studies have shown that dsRNA-mediated silencing of chitin synthesis-related genes in *Phakopsora pachyrhizi* [20] and *F. graminearum* [47] effectively reduces disease symptoms. Additionally, large-scale target screening remains critical for improving the efficacy of SIGS. For example, in *S. sclerotiorum*, only 11 of 58 tested dsRNAs successfully reduced lesion size by more than 50% [46]. These results highlight the necessity of large-scale screening approaches to identify effective fungal targets for SIGS.

In addition to designing dsRNAs that target a single essential gene, we also generated a chimeric Bcergi dsRNA that simultaneously targets *Bcerg1*, *Bcerg2,* and *Bcerg27* in *B. cinerea*. Surprisingly, the 270 bp *Bcerg1* dsRNA suppressed disease development more effectively than the 470 bp chimeric Bcergi dsRNA. This discrepancy likely stems from both fragment selection and overall dsRNA length. Previous studies have shown that simultaneously silencing functionally redundant genes such as *dicer1* and *dicer2* in *B. cinerea* or *cyp51A*, *B* and *C* in *F. graminearum* can reduce disease more efficiently than targeting a single gene [10, 11]. Nevertheless, those chimeric dsRNAs still affect only one critical step in the relevant pathway. In our study, *Bcerg1* encodes squalene epoxidase, an enzyme indispensable for the initial step of ergosterol biosynthesis and thus for fungal viability [48], whereas *Bcerg2* and *Bcerg27* act later in the pathway and appear less essential [49]. Consequently, dsRNA directed against *Bcerg1* alone produced the most significant reduction in lesion size (Fig. 5B, C). Furthermore, the chimeric Bcergi dsRNA contains only 150 bp of *Bcerg1* sequence and therefore generates fewer siRNAs than the 270 bp *Bcerg1* dsRNA. RT-qPCR confirmed that the 270 bp dsRNAs silenced their target more efficiently than the 470 bp chimeric dsRNA (Fig. 6C). Consistent with our results, dsRNAs longer than 400 bp have been reported to hinder uptake by *F. graminearum*, thereby weakening SIGS-mediated disease suppression [50]. Taken together, these findings indicate that both precise target selection and optimal dsRNA length are crucial for maximizing the efficacy of chimeric dsRNAs in plant disease control.

## Conclusions

In this study, we developed a pSIG system for scalable and highly efficient dsRNA production in the *E. coli* HT115 (DE3) strain. This system enables the rapid construction and production of chimeric dsRNAs that target multiple genes through a one-step GG cloning process. Owing to its high cloning efficiency, the entire workflow, from plasmid construction to dsRNA production, can be completed within four days. Using the pSIG system, we successfully produced 12 dsRNAs and a chimeric dsRNA showed that those targeting certain ergosterol biosynthesis genes significantly reduced *B. cinerea* disease infections. A shorter *Bcerg1* dsRNA targeting the key gene in the ergosterol biosynthesis pathway conferred stronger disease control than a longer chimeric dsRNA that simultaneously targets three ergosterol genes. In summary, the pSIG system offers an efficient alternative to in vitro dsRNA synthesis methods, producing sufficient dsRNA quantities for SIGS-based plant disease control applications.

## Methods

### Fungal growth and sporulation

The *Botrytis cinerea* strain B05.10 used in this study was obtained from a previous study [37]. The fungus was cultured on potato dextrose agar (PDA; Difco Laboratories, USA) at 25 °C and a 12-h light/dark cycle. For sporulation, cultures were transferred to an 18 °C growth chamber equipped with near-ultraviolet light [51]. Seven days later, the conidia were harvested by washing the plates with sterile Milli-Q water and filtering through two layers of sterile Miracloth. The conidia concentration was determined via a hemocytometer and adjusted to 2 × 10^4^ conidia/mL for the plant inoculation assays.

### Construction of pSIG plasmid vectors

The pSIG1 plasmid vector was constructed using the GG MoClo Toolkit (Addgene, USA) with modifications [27, 30]. Briefly, the T7 promoter and terminator sequences were PCR amplified with primers pSGB3_R_F and pSGB3_R_L (Supplementary Table 1). The PCR products were treated with DpnI, T4 polynucleotide kinase (PNK) and T4 DNA ligase (New England Biolabs, USA), and ligated into pICH47732, creating a plasmid with inverted T7 promoter and terminator sequences. Next, the T7 promoter and terminator were reamplified using primers BpiI_T7proter_B1_F/R and BpiI_T7proter_D2_F/R (Supplementary Table 1), and subsequently cloned into pICH41233 and pICH41276 using BpiI and T4 DNA ligases. The *SacB* gene was amplified from the pK18mobSacB plasmid that was gifted from Dr. Wen-Ling Deng, National Chung-Hsing University, Taiwan and was cloned into pICH47732 plasmid alongside the T7 promoter/terminator fragments, using BsaI and T4 DNA ligases to generate pSIG1. The pSIG2 plasmid was constructed by amplifying the *SacB* gene, T7 promoter, and terminator from pSIG1 using primers (Supplementary Table 1). The purified PCR products were assembled using GG cloning with BsaI and T4 DNA ligases.

### Selection of essential genes from *B. cinerea* and bioinformatics analyses

The target genes in *B. cinerea* B05.10 were selected based on the mode of action of fungicides as described by the Fungicide Resistance Action Committee (FRAC) [38]. The candidate genes included those involved in chitin biosynthesis (*Bcchs3a*, *Bcchs3b*, *Bcchs6*), ergosterol biosynthesis (*Bcerg1*, *Bcerg2*, *Bcerg24*, *Bcerg27*, *Bccyp51*), and melanin biosynthesis (*Bcpks12*, *Bcpks13*, *Bcbrn1*, *Bcbrn2*) (Table 1). Additional genes, *VPS51*, *DCTN1*, and *SAC1*, were selected on the basis of their involvement in vesicle trafficking [35]. The *GFP* gene was used as a negative control. DsRNA design against gene targets in the *B. cinerea* and prediction of off-target effects in humans were performed using the si-Fi software [39]. For each gene target, a 250 bp sequence predicted to generate the highest number of siRNAs was selected.

### Construction of the dsRNA producing plasmids

DNA fragments of target genes were PCR amplified from *B. cinerea* cDNA using the PCRBIO VeriFi™ Polymerase (PCR Biosystems, UK). GFP was PCR amplified from the pEKAR-G/R plasmid (Addgene #18,680) [52]. The primers used for these reactions contained BsmBI recognition sites at their N-terminus with overhangs complementary to GATC and AACG (Supplementary Table 1). The amplified DNA fragments were subsequently cloned into the pSIG1 plasmid via GG assembly using BsmBI (Thermo Fisher Scientific, USA) and T4 DNA ligase under the following thermocycler conditions: 37 °C for 20 s, followed by 26 cycles of 37 °C for 3 min and 16 °C for 4 min, and final steps of 50 °C for 5 min and 80 °C for 5 min. The GG products were transformed into *E. coli* HT115 (DE3) (a gift from Dr. Meng-Hsiao Meng, National Chung-Hsing University, Taiwan) using heat shock. Positive clones were selected on LB agar supplemented with 10% sucrose, 100 μg/mL carbenicillin and 25 μg/mL tetracycline, and confirmed via PCR and sequencing (Tri-I Biotech Inc., Taiwan).

To construct the plv2-dsErg2-R plasmid that was used to test autolysis in *E. coli* HT115 (DE3), the following steps were followed. First, a 250 bp segment of the *Bcerg2* gene was amplified from *B. cinerea* cDNA via PCR and cloned into the pSIG1 plasmid, generating the pSIG1-dsErg2 construct. Then, the pSIG1-dsErg2 endolysin gene (*R*), a dummy plasmid (pICH54022), and an end linker (pICH41780) were assembled into the MoClo Level 2 vector pAGM4723 using GG cloning with BpiI and T4 DNA ligase. The endolysin *R* gene was obtained from a previous study [36].

### Cloning and transformation efficiency in pSIG1 and *E. coli* HT115 (DE3)

To test the cloning efficiency in the pSIG1 plasmid and subsequent transformation in *E. coli* HT115 (DE3), 5 μL of an OD600 0.1 culture of *E. coli* HT115 (DE3) containing pSIG1 was spread onto LB agar with or without 10% sucrose. GFP and chimeric multi-fragment (VPS51, DCTN1, SAC1) inserts were subsequently cloned and inserted into pSIG1 via GG assembly. Colony PCR (primers Lv1_F and Lv1_R, Supplementary Table 1) was used to evaluate the efficiency across three independent experiments (10 colonies per experiment).

### In vivo dsRNA production

*E. coli HT115* (DE3) bacteria transformed with the dsRNA-producing plasmids were cultured overnight in 5 mL of LB supplemented with 100 μg/mL carbenicillin and 25 μg/mL tetracycline. Cultures were diluted into 20 mL of fresh LB and grown to an OD600 of 0.8. DsRNA production was induced with 1 mM IPTG for 6 h at 37 °C and shaking of the culture at 150 rpm. The bacteria were then centrifuged at 5000 rpm, and cell pellets were stored at −80 °C.

### Total RNA extraction and dsRNA purification

Total RNA was extracted from *E. coli* HT115 (DE3) using 1 mL of TRI Reagent (Sigma‒Aldrich, USA) per bacterial pellet, generated from a 10 mL culture as described above. After incubation at room temperature, 200 μL of 1-bromo-3-chloropropane (BCP) was added to separate the RNA-containing phase. The RNA was precipitated with 500 μL of 2-propanol, followed by washing with 75% ethanol (v/v). The RNA pellet was dissolved in 100 μL of RNase-free water, and the concentration was determined using an EzDrop spectrophotometer (Blue-Ray Biotech, Taiwan).

For dsRNA purification, 20 μg of total RNA was added to a mixture containing 2 μL of DNase I (New England Biolabs, USA), 5 μL of 10X DNase I buffer, and 1 μL of RNase T1 (Thermo Fisher Scientific, USA) in a final volume of 50 μL. This mixture was incubated at 37 °C for 45 min to degrade the DNA and single-stranded RNA. The dsRNA was cleaned and concentrated using an RNA Cleanup Kit (Geneaid Biotech, Taiwan). The quantity and purity of the dsRNA were assessed via spectrophotometry and gel electrophoresis.

For *E. coli* HT115 (DE3) carrying the autolysis plasmid plv2-dsErg2-R, RNA extractions were performed after inducing lysis via a freeze–thaw cycle. Following IPTG induction (1 mM IPTG in a 1 mL culture), the bacterial pellet was resuspended in 1 mL of RNase-free water, frozen at −80 °C, and thawed at 37 °C, with shaking at 100 rpm. The lysate was centrifuged, and the RNA in the supernatant was extracted and purified using an RNA Cleanup Kit (Geneaid Biotech, Taiwan). A control culture, processed identically but without the freeze–thaw step, was used for comparison. The RNA quality was validated via gel electrophoresis.

### Botrytis cinerea leaf inoculations

Inoculations with *B. cinerea* strain B05.10 were performed on detached leaves of 4-to-6 week-old *N. benthamiana* plants. A droplet containing 100 conidia was applied to each leaf. For each leaf, 1800 ng of GFP-targeting dsRNAs was applied to the left side, whereas 600 ng of *B. cinerea*-targeting dsRNAs was applied to the right side, maintaining the molar ratio of dsRNAs (

 ~ 15 pmol). The conidia and dsRNA mixtures were prepared in 1% Sabouraud Maltose Broth (SMB) (HiMedia Laboratories, India) with a final volume of 20 μL per droplet. To evaluate the chimeric RNA, 600 ng of *Bcerg1*, *Bcerg2*, or *Bcerg27* dsRNA was applied to the left side, whereas 1,080 ng of the chimeric Bcergi dsRNA was applied to the right side. Inoculated leaves were placed on plastic trays containing 1.5% (w/v) water agar (WA) and incubated at 25 °C. At three days post-inoculation (dpi), infected leaves were collected for RNA extraction and RT‒qPCR analysis. Lesion sizes were measured at four dpi using the Fiji software [53].

### RNA extractions from infected leaves and RT‒qPCR

Infected leaves were collected and stored at −80 °C until RNA extractions. The samples were ground via a BeadBug™−6 microtube homogenizer (Benchmark Scientific, USA) at 3800 rpm for 30 s, and this process was repeated three times. Total RNA was extracted via the taco™ mini Nucleic Acid Automatic Extraction System (GeneReach Biotechnology, Taiwan) with the taco™ Plant DNA/RNA Extraction Kit, according to the manufacturer’s instructions. RNA quality was assessed by 1.2% agarose gel electrophoresis, and RNA concentration was measured using an EzDrop spectrophotometer (EzDrop 1000, Blue-Ray Biotech, Taiwan). To remove residual DNA, the extracted nucleic acids were treated with DNase I (New England Biolabs, USA), according to the manufacturer’s protocol. Complementary DNA (cDNA) was synthesized from the purified RNA using the ExcelRT™ Reverse Transcription Kit II (SMOBIO Technology, Taiwan), according to the manufacturer’s guidelines. RT‒qPCR was performed using the qPCRBIO SyGreen Mix (PCR Biosystems, UK) in a CFX Connect Real-Time PCR Detection System (Bio-Rad, USA). The cycling conditions were set as follows: 40 cycles of 95 °C for 5 s and 60 °C for 30 s. The *B. cinerea* actin gene was amplified with primers Bcactin_qPCR_F and Bcactin_qPCR_R (Supplementary Table 1), whereas the target genes were amplified using gene-specific primers (Supplementary Table 1). Relative expression levels of target genes were calculated via the 2^−ΔΔCT^ method [54].

## Supplementary Information

Additional file 1

## Data Availability

All data are available upon request to L-HC (lhchen010@nchu.edu.tw). pSIG1 and pSIG2 plasmids are available on Addgene (addgene no. 226908 and 226909).
