# Supplementary material for: pSIG plasmids, MoClo-compatible vectors for efficient production of chimeric double-stranded RNAs in Escherichia coli HT115 (DE3) strain
Source: Plant Methods. 2025 Jul 11;21:96. doi: 10.1186/s13007-025-01413-5 (PMC12247436; doi:10.1186/s13007-025-01413-5)
Supplement: Supplementary file 1 — Additional file 1 [file 13007_2025_1413_MOESM1_ESM.docx]

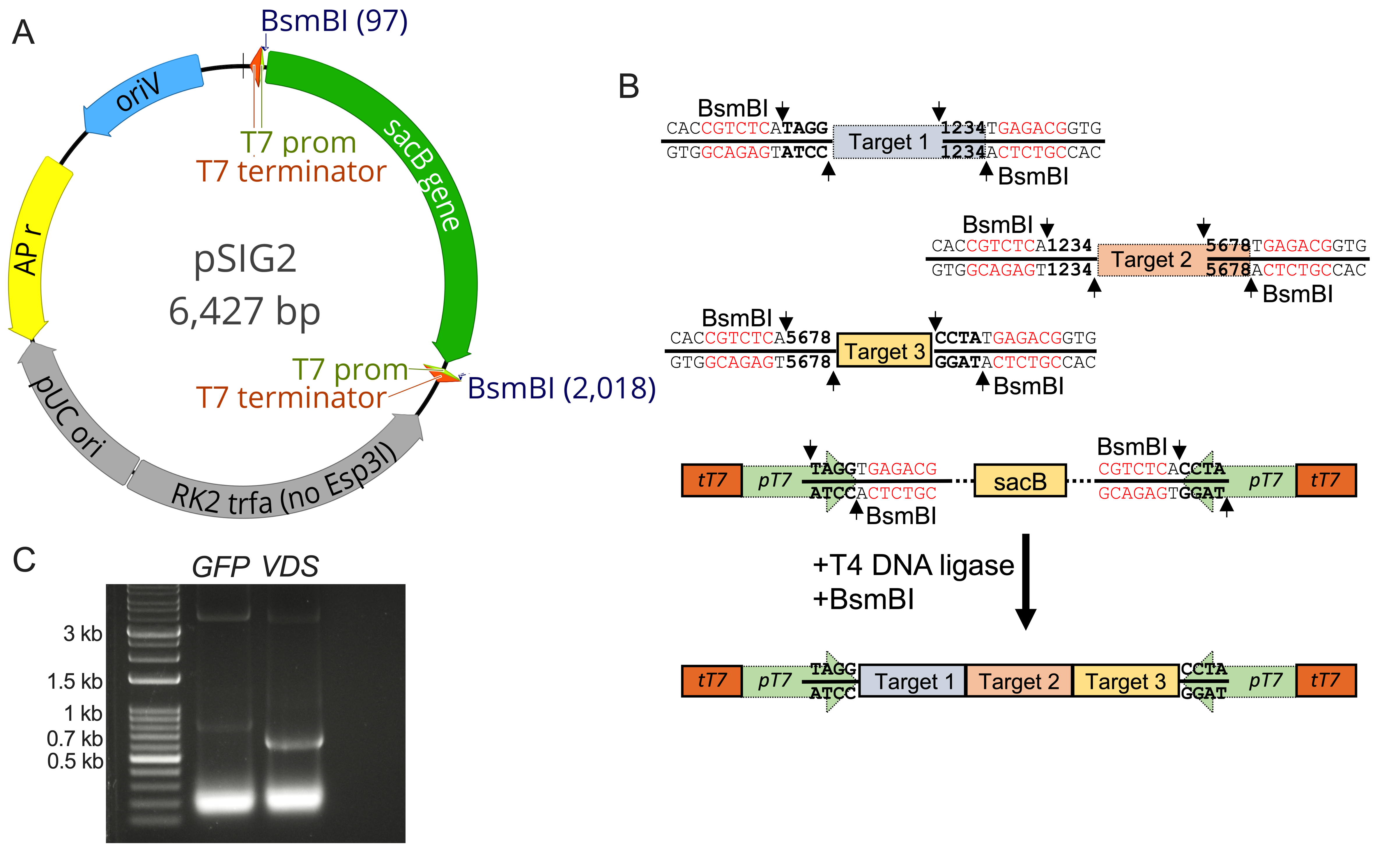


**Supplementary Figure 1.** Schematic of the pSIG2 plasmid and dsRNA production. **A** Diagram of the pSIG2 plasmid. Similar to pSIG1, the pSIG2 plasmid contains two T7 promoters, terminators, BsmBI recognition sites, and a *sacB* gene expression cassette within the pICH47732 backbone. **B** Strategy for cloning DNA fragments into the pSIG2 plasmid. DNA fragments were cloned into the pSIG2 plasmid using Golden Gate (GG) cloning with BsmBI and T4 DNA ligase. The BsmBI-generated overhangs in pSIG2 are identical and positioned in the two T7 promoters. **C** dsRNA production using the pSIG2 plasmid. GFP and VDS dsRNAs were visualized on an agarose gel following dsRNA production in *E. coli* HT115 (DE3).

**Supplementary Table 1.** Primer sequences used in this study

| Primer name | Primer sequence (5’ to 3’) | Description |
| --- | --- | --- |
| pSGB3_R_F | GGGGCCTCTAAACGGGTCTTGAGGGGTTTTTTGGCAATTGTCTTCTGCACGAAG | Generating T7 promoter  and terminator |
| pSGB3_R_L | AAGGGGTTATGCTAGTAATACGACTCACTATAGGGAGCGTGAGACCGTCACAGCTT | Generating T7 promoter  and terminator |
| BpiI_T7proter_B1_F | CCACAGAAGACGAGGAGCAAAAAACCCCTCAAGACCC | Cloning T7 promoter and terminator to pICH41233 |
| BpiI_T7proter_B1_R | CCACAGAAGACGAAGTACCTATAGTGAGTCGTATTACTAG | Cloning T7 promoter and terminator to pICH41233 |
| BpiI_T7proter_D2_F | CCACAGAAGACGAGCTTCCTATAGTGAGTCGTATTACTA | Cloning T7 promoter and terminator to pICH41276 |
| BpiI_T7proter_D2_R | CCACAGAAGACGAAGCGCAAAAAACCCCTCAAGACCC | Cloning T7 promoter and terminator to pICH41276 |
| BsaI_L1E1_Bsmb1_F | CACAGGTCTCGTACTCTAGTGAGACGAACCCATCACATATACCTGC | Cloning *sacB* gene |
| BsaI_L1E1_Bsmb1_R | CACAGGTCTCGAAGCCGTTTGAGACGGTACCCATCGGCATTTTCTTT | Cloning *sacB* gene |
| BsmBI_pSIG1_Bcerg1_F | ACACGTCTCGCTAGATGACTGTAGCCTTCAATGA | Constructing pSIG1_*Bcerg1* |
| BsmBI_pSIG1_Bcerg1_R | ACACGTCTCGCGTTCAATACAATTCCCTCCTCTT | Constructing pSIG1_*Bcerg1* |
| BsmBI_pSIG1_Bcerg2_F | ACACGTCTCGCTAGGTACTGAAGGACATACCGGA | Constructing pSIG1_*Bcerg2* |
| BsmBI_pSIG1_Bcerg2_R | ACACGTCTCGCGTTTCCAAAGTACTGCTAAGACC | Constructing pSIG1_*Bcerg2* |
| BsmBI_pSIG1_Bcerg24_F | ACACGTCTCGCTAGTATGCATTTGTCGCTCACCA | Constructing pSIG1_*Bcerg24* |
| BsmBI_pSIG1_Bcerg24_R | ACACGTCTCGCGTTCAAATACGCCGAGGGAAAGTG | Constructing pSIG1_*Bcerg24* |
| BsmBI_pSIG1_Bcerg27_F | ACACGTCTCGCTAGAACTTACAAGCTTGCAGAAA | Constructing pSIG1_*Bcerg27* |
| BsmBI_pSIG1_Bcerg27_R | ACACGTCTCGCGTTCCATATTATTCTTCCGCTGGT | Constructing pSIG1_*Bcerg27* |
| BsmBI_pSIG1_Bccyp51_F | ACACGTCTCGCTAGTGATGATTGCTCTTCTCATG | Constructing pSIG1_ *Bccyp51* |
| BsmBI_pSIG1_Bccyp51_R | ACACGTCTCGCGTTGGTGTGGTAACTTTGCGC | Constructing pSIG1_ *Bccyp51* |
| BsmBI_pSIG1_Bcchs3a_F | ACACGTCTCGCTAGGAGGCTATGATGAGCATAAG | Constructing pSIG1_*Bcchs3a* |
| BsmBI_pSIG1_Bcchs3a_R | ACACGTCTCGCGTTGTTGTAATCGCTGCTGTATG | Constructing pSIG1_*Bcchs3a* |
| BsmBI_pSIG1_Bcchs3b_F | ACACGTCTCGCTAGTCTTCGGCGCCGCGCTACTCGAAA | Constructing pSIG1_*Bcchs3b* |
| BsmBI_pSIG1_Bcchs3b_R | ACACGTCTCGCGTTCAAAAGCTCGGTGTGCCG | Constructing pSIG1_*Bcchs3b* |
| BsmBI_pSIG1_Bcchs6_F | ACACGTCTCGCTAGTCGGGGCTCCCACCATC | Constructing pSIG1_*Bcchs6* |
| BsmBI_pSIG1_Bcchs6_R | ACACGTCTCGCGTTCGACCAATAAATGAGAGGAG | Constructing pSIG1_*Bcchs6* |
| BsmBI_pSIG1_Bcpks12_F | ACACGTCTCGCTAGTCTTGCGGAACGCAATGTAC | Constructing pSIG1_*Bcpks12* |
| BsmBI_pSIG1_Bcpks12_R | ACACGTCTCGCGTTTTCGAGATACGATATCAAAC | Constructing pSIG1_*Bcpks12* |
| BsmBI_pSIG1_Bcpks13_F | ACACGTCTCGCTAGCAGCGCCATTGGTACGAG | Constructing pSIG1_*Bcpks13* |
| BsmBI_pSIG1_Bcpks13_R | ACACGTCTCGCGTTTATACTTTCCTTGTGTGCAG | Constructing pSIG1_*Bcpks13* |
| BsmBI_pSIG1_Bcbrn1_F | ACACGTCTCGCTAGGCAATGGGGTAAACTCGATAT | Constructing pSIG1_*Bcbrn1* |
| BsmBI_pSIG1_Bcbrn1_R | ACACGTCTCGCGTTCTCAATGGCACCCTTAGAAC | Constructing pSIG1_*Bcbrn1* |
| BsmBI_pSIG1_Bcbrn2_F | ACACGTCTCGCTAGAGCGGAAAAGGTTGTCAAG | Constructing pSIG1_*Bcbrn2* |
| BsmBI_pSIG1_Bcbrn2_R | ACACGTCTCGCGTTAGCTTGTTGAGCAACAAAG | Constructing pSIG1_*Bcbrn2* |
| BsmBI_pSIG1_ eGFP_F | ACACGTCTCGCTAGATGGTGAGCAAGGGCGAG | Constructing pSIG1_ *eGFP* |
| BsmBI_pSIG1_ eGFP_R | ACACGTCTCGCGTTCTTGTACAGCTCGTCCATG | Constructing pSIG1_ *eGFP* |
| BsmBI_pSIG2_ eGFP_F | ACAGAAGACGAGCTTCCTATAGTGAGTCGTATTACTAGCA | Constructing pSIG2_ *eGFP* |
| BsmBI_pSIG2_ eGFP_R | ACACGTCTCGTAGGCTTGTACAGCTCGTCCATGC | Constructing pSIG2_ *eGFP* |
| BsmBI_pSIG1_ BcVPS51-1_F | ACACGTCTCGCTAGTTCGTTCCAGGAGTTACACG | Constructing pSIG1_ *BcVPS51+DCTN1+SAC1* |
| BsmBI_pSIG2_ BcVPS51-1_F | ACACGTCTCGTAGGTTCGTTCCAGGAGTTACACGC | Constructing pSIG2_ *BcVPS51+DCTN1+SAC1* |
| BsmBI_pSIG1_ BcVPS51-1_R | ACACGTCTCGTGATGAGACAAGTGAGAGTCCA | Constructing pSIG1_ *BcVPS51+DCTN1+SAC1* |
| BsmBI_pSIG1_ BcDCTN1-2_F | ACACGTCTCGATCATTCGTGCTCTCGTCGAAAGC | Constructing pSIG1_ *BcVPS51+DCTN1+SAC1* |
| BsmBI_pSIG1_ BcDCTN1-2_R | ACACGTCTCGCCAGCTTACAACTGTGCTCT | Constructing pSIG1_ *BcVPS51+DCTN1+SAC1* |
| BsmBI_pSIG1_ BcSAC1-3_F | ACACGTCTCGCTGGACGTTGTTCAAAGTGCAGTG | Constructing pSIG1_ *BcVPS51+DCTN1+SAC1* |
| BsmBI_pSIG1_ BcSAC1-3_R | ACACGTCTCGCGTTCCTTCAATGCTGCTGTAGAAG | Constructing pSIG1_ *BcVPS51+DCTN1+SAC1* |
| BsmBI_pSIG2_ BcSAC1-3_R | ACACGTCTCGTAGGCCTTCAATGCTGCTGTAGAAGC | Constructing pSIG2_ *BcVPS51+DCTN1+SAC1* |
| BsmBI_pSIG1_Bcerg1-1_i_F | ACACGTCTCGCTAGACTTTCTCCATCCAACATCC | Constructing pSIG1_ *Bcergi* |
| BsmBI_pSIG1_Bcerg1-1_i_R | ACACGTCTCGTGTGGATCATTAGCTGCAAA | Constructing pSIG1_ *Bcergi* |
| BsmBI_pSIG1_Bcerg2-2_i_F | ACACGTCTCGCACACTGATGATTATTTCCACATC | Constructing pSIG1_ *Bcergi* |
| BsmBI_pSIG1_Bcerg2-2_i_R | ACACGTCTCGCAAGAGCGAAACAGGCCG | Constructing pSIG1_ *Bcergi* |
| BsmBI_pSIG1_Bcerg27-3_i_F | ACACGTCTCGCTTGGTTATCACCAAACCTCAACT | Constructing pSIG1_ *Bcergi* |
| BsmBI_pSIG1_Bcerg27-3_i_R | ACACGTCTCGCGTTAACGTAATGTCCGAAAACGT | Constructing pSIG1_ *Bcergi* |
| Lv1_F | GAACCCTGTGGTTGGCATGCACATAC | Confirming positive clony |
| Lv1_R | CTGGTGGCAGGATATATTGTGGTG | Confirming positive clony |
| Bcerg1_qPCR_F | AAGCCGCCAGTGAAGAAA | Checking *Bcerg1* expression |
| Bcerg1_qPCR_R | CTTGTTGGGCAAGTGCATAAG | Checking *Bcerg1* expression |
| Bcerg2_qPCR_F | CGACTCAACTCCATGATCTCTC | Checking *Bcerg2* expression |
| Bcerg2_qPCR_R | TGCTTCTCACTCAGTTCATCC | Checking *Bcerg2* expression |
| Bcerg27_qPCR_F | ACAATGGATGGTCTCCGATTAC | Checking *Bcerg27* expression |
| Bcerg27_qPCR_R | GACCCTTCCTTCTGACTCAATG | Checking *Bcerg27* expression |
| Bcactin_qPCR_F | TGCTCCAGAAGCTTTGTTCCAA | Checking *Bcactin* expression |
| Bcactin_qPCR_R | TCGGAGATACCTGGGTACATAG | Checking *Bcactin* expression |
|  |  |  |
